# Pathology and Treatment of Milk-Sickness

**Published:** 1867-04

**Authors:** W. B. Miller

**Affiliations:** Calhoun, Ky.


### ARTICLE II.

*Pathology and Treatment of Milk-sickness.* By W. B. Miller, M. D., of Calhoun, Ky.

We have no positive knowledge of the exciting cause of this disease. Medical talent has exhausted the field of conjecture, and left nothing new to advance, except an undoubted solution of the problem.

Some have found this protean entity in various productions of the vegetable kingdom—generally in the class of acro-narcotic poisons; but are met with the objection, that the habits of the vegetable flora are different—occupying extensive ranges of country, while the disease in question prevails in areas, limited and sharply defined. In other words, that the effect, milk-sickness, should be coëqual with the cause, vegetable poison. Others find this ignis fatuus in a marsh, and insist upon its miasmatic origin, in as much as it is most prevalent in the miasmatic season, and is attended with gastric and hepatic lesion, in common with paludal affections; but this view is liable to the fatal objection, that a large majority of the cases of milk-sickness are destitute of the sign and seal of marsh poison—periodicity. Another class derive the cause from mineral strata, alleging, with apparent plausibility, that the geological distribution of certain mineral poisons corresponds with the geographical range of milk-sickness; that the pathological phenomena of slow mineral poison are so nearly analogous to those of the so-called milk-sickness, as to point directly to a common origin. To the last, the reply is, that geological and geographical concurrence, if established, might have no relation as cause and effect, and that their analogy is too discrepant to pass for identity.

The pathological history and therapeutic treatment of the disease offers a more hopeful and profitable field of investigation; at least, we are not left to the total darkness of blind hypothesis, but have some established facts upon which to base theory, and from which to deduce practice.

Dr. J. M. Johnson, in an able and elaborate article, published in this Journal, has given so graphic a delineation of the disease, that it would be useless reiteration to recapitulate the symptoms.

All observers agree that, in the incubative stage, the characteristic symptoms are nervous and muscular debility. In the primary stage of full development, we have augmented derangement of nervous and muscular function. At this period, I have rarely found evidence of organic lesion; and I think the subsequent symptoms which pertain to this affection, *per se*, may, without violence, be ascribed to local suppression of nerve power. In other words, paralysis of the small intestines is the center around which revolve the varied phenomena of the disease.

We invariably find the small bowels torpid: indeed, so utterly deficient in excitability, that they fail to respond to the powerful stimulous of acrid purgatives; and so destitute of tone, that they speedily become distended with their fecal and gaseous contents. That the cerebro-spinal and vascular systems are only secondarily involved, is manifest from their comparatively slight degree of disturbance, except, indeed, in the ultimate stage of severe cases for which the toxical properties of the blood derived from absorption, deficient depuration, and perhaps occasional complicity of the disease with marsh poison, may, in part, account.

The concentrated influence of the poison upon the small bowels is probably because the stomach and duodnum being capacious, and frequently washed with draughts of water, to allay attending thirst, the poison mixed with glutinous matter is swept into the intestines, where it is liable to lodge and accumulate, in consequence of their small caliber and tortuous position.

The virus being in immediate contact with sympathetic nerve capillaries while exercising a direct influence in lessening the muscular irritability and tone of the bowel, reflects its sedative effects upon the corresponding nerve center, diminishing its capacity for the reception or conveyance of impressions. The reverse of this condition obtains in the superior portion of the intestinal tube, evidenced by gastric irritation, augmented mucus, and billiary secretions, and excessive emesis—illustrating a physio-pathological law—that suppressed function is followed by vicarious effort at compensation. Warned by the sentinal nerves, still competent for the duty, the stomach and duodenam are goaded to powerful and spasmodic effort for casting out the offending cause.

An orgasm in the stomach and duodenam that is sufficient to produce muscular contraction to the extent of prolonged and severe vomitive effort, should be ample to produce stricture in the more sensitive and contracted volvular aperture, at the inferior orifice of the duodenam; and, accordingly, we so find it to a degree that occasionally precludes the passage of even bland fluids to say nothing of acrid ingesta. The rectum and sphincter ani receiving their supply from the cerebro spinal system retain more of their normal condition. The superior and inferior parts of the alimentary canal may be compared to positive and negative electric poles, and the intervening poison to the non-conducting substance that bars equilibrium. The small bowels, containing the poison, may be likned to a closed sac, tyed at one extremity by stricture, and at the other by torpor.

The mucus lining of the intestinal tube receives its nerves almost exclusively from the ganglionic system; and since the intestinal ganglia do not yield the nervous supply necessary for the intestinal contraction, except by their centripetal nerves through stimuli of substances in the intestinal canal (Gross,) our remedies should be applied accordingly. It is clear, however, that, before we can obtain access to the point of invasion, we must allay gastric erethism and duodenal stricture. This we may accomplish by counter irritation and the internal or endermic administration of Morphia.

The next disderatum is a stimulant cathartic of special nerve affinity, and of sufficient energy not only to vanquish the specific inertia induced by milk-poison, but to excite the bowels to vigorous effort for the prompt expulsion of the morbific cause.

The Oil of Turpentine, in the combination recommended by Dr. Johnson, to wit:—Ven Turpentine, Castor Oil, and Comp. Spts. Lavender, fills the indications precedent, and hence its remarkable efficacy in the treatment of the disease.

My own clinical experience for twenty years, and that of my father and preceptor, Dr. W. Miller, for a much longer period, fully coincides with Dr. Johnson's roport of its remedial powers. I have rarely had occasion to resort to any other agent for its successful treatment. When called to advanced cases, where the vital powers were rapidly sinking, in connection with the above remedy, I have used actively the diffuseble stimuli, with Quinia, Strychnia, and suitable aliment to render permanent advantages thus obtained. The calorific function in this, as in nearly all other cases of poisoning, is remarkably modified, and must be sedulously guarded and assisted, when requisite by artificial means.

A majority of practitioners in this section rely upon opiates, stimulants, and the persevering use of alkaline effervescing salts, in the treatment of milk-sickness. I have never tested this treatment, but with my pathological views, can readily credit the report of its general success. Opium, in allaying spasm, arrests the regurgitive current of bile through the stomach and æsophigus, and returns it to its natural channel. Bile is a peculiar and appropriate intestinal stimulant, distilled for that purpose from natures alembic, and acting in conjunction with alcoholic reinforcement, arouses intestinal sensibility, while the purgative property of the salts completes the curative movement by aiding in the expulsion of the poison.

It is probable that alkalies, having numerous chemical affinities, may enter into a more or less antidotal combination with the poison. All know that acids, which are always in excess in effervescing mixtures, have been recommended by high authority, and used with undoubted success, as a prophylactic to lead poison.

